# Supplementary material for: Contribution of Influenza Viruses, Other Respiratory Viruses and Viral Co-Infections to Influenza-like Illness in Older Adults
Source: Viruses. 2022 Apr 12;14(4):797. doi: 10.3390/v14040797 (PMC9024706; doi:10.3390/v14040797)
Supplement: Supplementary file 1 [file viruses-14-00797-s001.zip › viruses-1653802-supplementary.pdf]

viruses

MDPI

Supplementary Materials

Contribution of Influenza Viruses, Other Respiratory Viruses and Viral Co-Infections to Influenza-like Illness in Older Adults

Citation: Kaaijk, P.; Swaans, N.; Nicolaie, A.M.; Bruin, J.P.; van Boxtel, R.A.J.; de Lange, M.M.A.; Meijer, A.; Sanders, E.A.M.; van Houten, M.A.; Rots, N.Y.; et al. Contribution of Influenza Viruses, Other Respiratory Viruses and Viral Co-Infections to Influenza-like Illness in Older Adults. *Viruses* **2022**, *14*, 797. https://doi.org/10.3390/v14040797

Academic Editor: Ayato Takada

Received: 10 March 2022
Accepted: 7 April 2022
Published: 12 April 2022

Publisher's Note: MDPI stays neutral with regard to jurisdictional claims in published maps and institutional affiliations.

Copyright: © 2022 by the authors. Licensee MDPI, Basel, Switzerland. This article is an open access article distributed under the terms and conditions of the Creative Commons Attribution (CC BY) license (https://creativecommons.org/licenses/by/4.0/).

Table S1. Viruses detected in naso- and oropharyngeal swabs.

			**ILI events**	**ILI at 14d**	**ILI recovery events at 8 wk**	**Asymptomatic control events**	**Asymptomatic at 14 d**
			n (%)	*n* (%)	*n* (%)	*n* (%)	*n* (%)
Subjects			252	252	252	205	205
Events			254	254	254	205	203
Missing			-	4 (1.6%)	10 (3.9%)	-	-
No 14 days visit			-	8 (3.1%)	-	-	2 (1.0%)
No recovery visit			-	-	11 (4.3%)	-	-
No sample: 2nd ILI			-	1 (0.4%)	1 (0.4%)	-	-
Excluded from analysis[a]			-	13 (5.1%)	22 (8.7%)	-	2 (1.0%)
Any virus[b]			200 (78.7%)	73 (28.7%)	37 (14.60%)	28 (13.7%)	29 ((14.1%)
Influenza virus			100 (39.4%)	15 (5.9%)	4 (1.2%)	1 (0.5%)	2 (1.0%)
	-A		76 (76.0%)	14 (93.3%)	3 (75.0%)	0 (0%)	2 (100%)
		A (H1N1)pdm09	8 (10.5%)	0 (0%)	0 (0%)	-	
		A(H3N2)	68 (89.5%)	14 (100%)	2 (66.6%)	-	2 (100%)
		- 3C.2a	-39 (57.4%)				-2 (100%)
		- 3C.3b	-22 (32.4%)				
		- Not typable[c]	- 7 (10.3%)		1 (33.3%)		
	-B		24 (24%)	1 (6.7%)	1 (25.0%)	1 (100%)	0 (0%)
		B Victoria-like	0 (0%)			1 (100%)	-
		B Yamagata-like	24 (100%)	1 (100%)		0 (0%)	-
hMPV			16 (6.3%)	3 (1.2%)	2 (0.8%)	0 (0%)	2 (1.0%)
Coronavirus			25 (9.8%)	15 (5.9%)	13 (5.1%)	8 (3.9%)	5 (2.4%)
	-229E		9 (36%)	7 (46.7%)	5 (38.5%)	2 (25%)	1 (20.0%)
	-OC43		12 (48%)	5 (33.3%)	6 (46.2%)	1 (12.5%)	2 (40.0%)
	-NL63		2 (8%)	2 (13.3%)	1 (7.7%)	3 (37.5%)	0 (0%)
	-HKU1		2 (8%)	1 (6.7%)	1 (7.7%)	2 (25%)	2 (40.0%)
Rhinovirus			44 (17.3%)	25 (9.8%)	16 (6.3%)	17 (8.3%)	12 (5.9%)
RSV			17 (6.7%)	4 (1.6%)	0 (0%)	0 (0%)	3 (1.5%)
	-A		8 (47.1%)	2 (50%)	0 (0%)	0 (0%)	0 (0%

	-B			9 (52.9%)		2 (50%)		0 (0%)		0 (0%)		3 (100%)
Parainfluenza virus			11 (4.3%)		8 (3.1%)		1 (0.4%)		2 (1.0%)		5 (2.4%)	
	-1			0 (0%)		0 (0%)		0 (0%)		0 (0%)		0 (100%)
	-2			3 (27.3%)		3 (37.5%)		0 (0%)		0 (0%)		0 (100%)
	-3			4 (36.4%)		4 (50%)		1 (100%)		1 (50%)		3 (60.0%)
	-4			4 (36.4%)		1 (12.5%)		0 (0%)		1 (50%)		2 (40%.0)
Bocavirus			0 (0%)		3 (1.2%)		1 (0.4%)		0 (0%)		1 (0.5%)	
Adenovirus			0 (0%)		7 (2.8%)		2 (0.8%)		0(0%)		1 (0.5%)	

All viruses were detected by MLPA. In addition, influenza A (H3N2) was subtyped by sequencing. Abbreviations: 14d, 14 days; 8 wk, 8 weeks; hMPV, human metapneumovirus; ILI, influenza-like illness; RSV, respiratory syncytial virus; [a]Excluded from analysis includes ILI events with incomplete visits, no swabs available and drop-outs; [b]Any virus: includes ILI events whereby corresponding samples contained multiple viruses (co-infections); [c]Not typable: % could not be further characterized due to sequencing failure as a result of low viral load.

Table S2. Occurrence of virus co-infections.

	**2012–2013 Co-infections (*n*)**	**2014–2015 Co-infections (*n*)**
Influenza virus-Rhinovirus	5	3
Influenza virus-Coronavirus	2	3
Influenza virus-hMPV	0	0
Influenza virus-RSV	2	3
Rhinovirus-Coronavirus	1	1
Rhinovirus-RSV	2	0
Rhinovirus- Parainfluenza virus	3	1
Coronavirus-RSV	1	0
Coronavirus-hMPV	1	0
RSV-Parainfluenza virus	1	0
hMPV-Parainfluenza virus	1	0
Coronavirus-Adenovirus	1	0
Influenza virus-Bocavirus	1	0
Influenza virus-Coronavirus-RSV	0	1

Abbreviations: hMPV, human metapneumovirus; RSV, respiratory syncytial virus.
